# A novel machine learning model for class III surgery decision

**DOI:** 10.1007/s00056-022-00421-7

**Published:** 2022-08-26

**Authors:** Hunter Lee, Sunna Ahmad, Michael Frazier, Mehmet Murat Dundar, Hakan Turkkahraman

**Affiliations:** 1 Department of Orthodontics and Oral Facial Genetics, Indiana University School of Dentistry, 1121 West Michigan Street, 46202 Indianapolis, IN USA; 2 Indiana University School of Dentistry, Indianapolis, IN USA; 3 Department of Computer and Information Science, School of Science, Indiana University Purdue University Indianapolis, Indianapolis, IN USA

**Keywords:** Artificial intelligence, Orthognathic surgery, Computer-assisted decision making, Dentofacial deformities, Logistic models, Künstliche Intelligenz, Kieferorthopädische Chirurgie, Computergestützte Entscheidungsfindung, Dentofaziale Deformitäten, Logistische Modelle

## Abstract

**Purpose:**

The primary purpose of this study was to develop a new machine learning model for the surgery/non-surgery decision in class III patients and evaluate the validity and reliability of this model.

**Methods:**

The sample consisted of 196 skeletal class III patients. All the cases were allocated randomly, 136 to the training set and the remaining 60 to the test set. Using the test set, the success rate of the artificial neural network model was estimated, along with a 95% confidence interval. To predict surgical cases, we trained a binary classifier using two different methods: random forest (RF) and logistic regression (LR).

**Results:**

Both the RF and the LR model showed high separability when classifying each patient for surgical or non-surgical treatment. RF achieved an area under the curve (AUC) of 0.9395 on the test set. 95% confidence intervals were computed by bootstrap sampling as lower bound = 0.7908 and higher bound = 0.9799. On the other hand, LR achieved an AUC of 0.937 on the test set. 95% confidence intervals were computed by bootstrap sampling as lower bound = 0.8467 and higher bound = 0.9812.

**Conclusions:**

RF and LR machine learning models can be used to generate accurate and reliable algorithms to successfully classify patients up to 90%. The features selected by the algorithms coincide with the clinical features that we as clinicians weigh heavily when determining a treatment plan. This study further supports that overjet, Wits appraisal, lower incisor angulation, and Holdaway H angle can be used as strong predictors in assessing a patient’s surgical needs.

## Abstract (de)

**Zielsetzung:**

Primäres Ziel dieser Studie war es, ein neues Machine-Learning-Modell für die Entscheidung Operation vs. nichtoperative Behandlung bei Klasse-III-Patienten zu entwickeln und die Validität und Reliabilität dieses Modells zu bewerten.

**Methoden:**

Die Stichprobe bestand aus 196 Patienten der skelettalen Klasse III. Alle Fälle wurden randomisiert einer Gruppe zugewiesen, 136 der Trainingsgruppe und die übrigen 60 der Testgruppe. Anhand des Testsatzes wurde die Erfolgsquote des künstlichen neuronalen Netzes mit einem Konfidenzintervall von 95% abgeschätzt. Zur Prädiktion chirurgischer Fälle wurde ein binärer Klassifikator mit 2 unterschiedlichen Methoden trainiert: Random Forest (RF) und logistische Regression (LR).

**Ergebnisse:**

Sowohl das RF- als auch das LR-Modell zeigten eine hohe Trennschärfe bei der Klassifizierung der einzelnen Patienten für eine chirurgische bzw. eine nichtchirurgische Behandlung. RF erreichte eine AUC („area under the curve“) von 0,9395 in der Testgruppe. Die 95%-Konfidenzintervalle wurden mittels Bootstrap-Stichproben als untere Grenze = 0,7908 und obere Grenze = 0,9799 berechnet. Andererseits erreichte LR eine AUC von 0,937 in der Testgruppe. Die 95%-Konfidenzintervalle wurden durch Bootstrap-Sampling als untere Grenze = 0,8467 und obere Grenze = 0,9812 berechnet.

**Schlussfolgerungen:**

Mithilfe von RF- und LR-Modellen für maschinelles Lernen lassen sich genaue und zuverlässige Algorithmen erstellen, die Patienten in bis zu 90% der Fälle erfolgreich klassifizieren. Die von den Algorithmen ausgewählten Merkmale stimmen mit den klinischen Merkmalen überein, die wir als Kliniker bei der Festlegung eines Behandlungsplans stark gewichten. Diese Studie belegt außerdem, dass Overjet, Wits-Appraisal, die Angulation der unteren Inzisiven und der Holdaway-H-Winkel als starke Prädiktoren für die Beurteilung des Operationsbedarfs eines Patienten verwendet werden können.

## Introduction

The most important part of orthodontic treatment is a proper diagnosis and the establishment of a treatment plan [1]. A proper diagnosis defines the problems of the patient so that a problem list can be identified. Once the diagnosis is made, clinicians should establish treatment goals to address the identified problems. There are many instances in which orthodontic therapy alone can be used to camouflage skeletal discrepancies with dental compensations. Other times it is necessary for the clinician to include orthognathic surgery as a part of the treatment plan. The pivotal part of treatment planning is the decision about whether orthognathic surgery is needed. Various factors such as desired profile changes, size of the upper airway, crowding, incisor position, and long-term stability must be taken into consideration [2]. Previous studies have identified several cephalometric measurements that can be used to help distinguish between surgical and non-surgical treatment with specificity as high as 90% [2–6]. The importance of this decision must be seriously considered in order to protect patients from unnecessary risks that may lead to complications such as infection, postoperative malocclusion, hemorrhage, bad splits, inferior alveolar nerve injury, and irreversible treatment such as extractions [7].

Expert clinicians have been sculpted by their education and clinical experiences to develop their treatment philosophies. It is very difficult to develop this process in a short amount of time for inexperienced clinicians. Treatment planning is a complex process in which diagnostic data is organized and combined with background knowledge and clinical experience that simply cannot be standardized into a formula [8]. An inexperienced orthodontist would benefit greatly if an artificial intelligence (AI) system existed that can be used to supplement this gap in experience. Moreover, AI systems may act as a complementary method that aids in decision-making, like a second opinion. AI systems are not new to the field of dentistry [9]. Over the last two decades, AI models have been generated to help with endodontic diagnosis [10], radiographic diagnosis [11], and to determine orthodontic treatment needs [12]. More recently in orthodontics, a variety of methods have been studied in the construction of an AI system that can support diagnosis, treatment planning, and planned tooth movement [13–15].

Among the methods of constructing an AI system, supervised machine learning is a method that allows computers to mimic the expert thought process and rationale in decision making. Supervised learning methods use a training dataset usually retrospectively collected from electronic archives and contains a set of dependent and independent variables for each case [16]. In the context of the proposed project, the dependent variable was the diagnostic decision assigned to each case by the practicing orthodontist, and independent variables were demographic data and the measurements obtained from diagnostic records. Two main categories of supervised learning techniques involve discriminative and generative models. Discriminative models learn a mapping between input values and corresponding output values for all cases in the training set by optimizing linear or nonlinear discriminant functions [17]. Among the most popular algorithms in this category are logistic regression [18], support vector machines [19], and neural networks [20]. On the other hand, generative models estimate the underlying probability distributions for each class and renders classification based on Bayes’ rule [17]:

$$P(A|B)=\frac{P(B|A)\times P(A)}{P(B)}$$

The current project required a binary decision which leads to two classes: surgery vs. non-surgery.

There is currently only one other study that has used machine learning to develop and evaluate a model to incorporate this technology in the treatment planning of orthognathic surgery cases [21]. However, this study only included a limited number of cephalometric values and additional objective indexes. It was our goal to increase the number of cephalometric values in the input data set to expand the search for causal relationships between the independent and dependent variables. We also took into consideration the patient’s subjective desire to seek surgical treatment for esthetic reasons. It was our aim to develop a new machine learning model for surgery/non-surgery decision in class III patients and evaluate the validity and reliability of this novel model.

## Materials and methods

### Ethical statement

This project was submitted for review to the Indiana University Institutional Review Board and approved (March 03, 2021, #10220).

### Study design

This was a retrospective study, and the sample consisted of 196 skeletal class III patients who visited the Department of Orthodontics and Orofacial Genetics, Indiana University. The subjects included in the study had a negative ANB value and a Wits analysis that measured less than negative one millimeter. The exclusion criteria for the study included subjects with missing teeth except for third molars, malformed teeth, craniofacial anomalies such as cleft palate, and patients with a documented anterior functional shift.

A full set of orthodontic records was collected for each. Treatment plans were decided by 1 orthodontic resident and 2 faculty orthodontic specialists. All 3 clinicians were blinded against the others’ decisions, when the initial treatment decision was first made. A complete agreement was reached in 167 out of 196 cases (85%) during this blinded initial treatment decision process. The remaining 29 cases (15%) were re-evaluated for a second time as a group, and a final treatment decision was made by complete agreement of all the examiners.

**Fig. 1:**
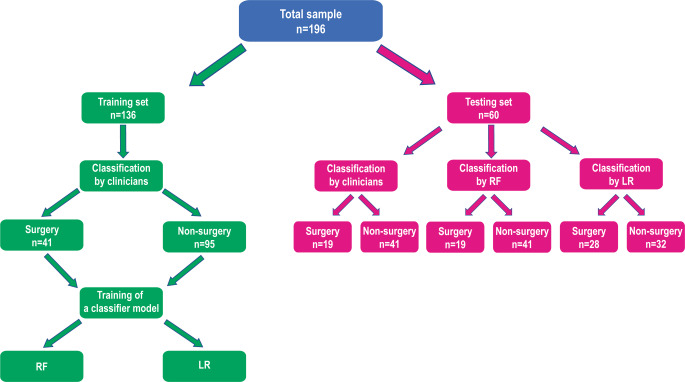
Flow chart representing the group allocation, training and testing processes. *RF* random forest, *LR* logistic regression Flussdiagramm, in dem die Prozesse der Gruppeneinteilung, der Trainings und der Tests dargestellt sind. *RF* Random Forest, *LR* logistische Regression

A flow chart representing the group allocation, training, and testing processes is shown in Fig. 1. All the cases were allocated randomly, 136 to the training set and the remaining 60 to the test set. Randomization to the training and test sets was stratified by age, gender, and surgery, with proportional allocation to training/test sets based on those three factors. The test set was not used for the model construction and only used to evaluate the validity of the constructed model. To assess the reliability of the constructed model, 50 cases from the training set were used. The input values were obtained from 46 cephalometric measurement values (Table 1) and 7 additional indexes (Table 2). Categorical variables (“Sex at birth”, “Chief complaint” and “Molar classification”) in the data were first converted into one-hot encoding vectors. With this extension the number of features increased from 53 to 60. All feature values were normalized to between 0 and 1. A regularization constant that adjusts the tradeoff between regularization and empirical error was set to 0.5. Tracing and measurement of the lateral cephalogram for each patient were performed digitally by one investigator (H.L.) using Dolphin Imaging Version 12.0.09.39 (Patterson Dental Supply Inc., Chatsworth, CA, USA). Of the 196 included patients, 20 were randomly chosen and the cephalometric radiographs were traced again by the same examiner to measure method error of the tracing.

**Table 1:** Description of the lateral cephalometric data Beschreibung der lateralen kephalometrischen Daten

Measurement	Description
*Maxilla to cranial base*	
SNA (°)	Angle between sella, nasion and A point
SN-palatal plane (°)	Angle between sella-nasion and palatal plane
Occlusal plane to SN (°)	Angle between sella-nasion and occlusal plane
A‑N perpendicular (mm)	Distance from A point to the plane drawn perpendicularly from nasion to the Frankfort horizontal plane
*Mandible to cranial base*	
SNB (°)	Angle between sella, nasion and B point
SNPg (°)	Angle between sella, nasion and pogonion
FMA (FH-MP) (°)	Angle between Frankfort horizontal plane and gonion-gnathion
SN-MP (°)	Angle between sella-nasion and gonion-gnathion
Mandibular plane to occlusal plane (°)	Angle between mandibular plane and occlusal plane
B‑N perpendicular (mm)	Distance from B point to the plane drawn perpendicularly from nasion to the Frankfort horizontal plane
Pog‑N perpendicular (mm)	Distance from pogonion to the plane drawn perpendicularly from nasion to the Frankfort horizontal plane
Y‑Axis (SGn-SN) (°)	Angle between the sella-gnathion and sella-nasion
*Maxilla to mandible*	
ANB (°)	Angle between sella, nasion and B point
Palatal plane to mandibular plane angle (PP-MP) (°)	Angle between the ANS-PNS and Go-Gn lines
Wits appraisal (mm)	Distance between the projections of points A and B on the occlusal plane
Maxillary length (ANS-PNS) (mm)	Linear distance from anterior and posterior nasal spines
Mandibular length (Co-Gn) (mm)	Linear distance from condylion to gnathion
*Cranial base*	
Ba-S‑N (°)	Angle between basion, sella and nasion
*Upper incisors to maxilla*	
U1-SN (°)	Angle between long axis of upper incisor and sella-nasion
U1-NA (°)	Angle between long axis of upper incisor and nasion-A point
U1-NA (mm)	Distance from upper incisor tip to nasion-A point line
U1-palatal plane (°)	Angle between long axis of upper incisor and palatal plane
U1 protrusion (U1-APo) (mm)	Distance from upper incisor tip to A point-pogonion line
*Lower incisors to mandible*	
L1-MP (°)	Angle between long axis of lower incisor and mandibular plane
L1-NB (°)	Angle between long axis of lower incisor and nasion-B point
L1-NB (mm)	Distance from lower incisor tip to nasion-B point line
L1 protrusion (L1-APo) (mm)	Distance from lower incisor tip to A point-pogonion line
Holdaway ratio	Ratio of the linear distance from the labial surface of the mandibular central incisor to the NB line over the linear distance of the chin to the same line
*Incisors to each other*	
Interincisal angle (U1-L1) (°)	Angle between the long axes of upper and lower incisors
Overjet (mm)	Vertical overlap between upper and lower incisors
Overbite (mm)	Sagittal overlap between upper and lower incisors
*Soft tissue*	
Upper lip to E‑plane (mm)	Distance from the upper lip stomion to the E‑plane
Lower lip to E‑plane (mm)	Distance from the lower lip stomion to the E‑plane
ILG (mm)	Distance between the upper and lower lip at rest
Nasolabial angle (Col-Sn-UL) (°)	Angle between columella, subnasale and upper lip
H‑Angle (Pg′UL-Pg′Na′) (°)	Angle between soft tissue nasion, pogonion and upper lip
UFH (G′-Sn′) (mm)	Soft tissue upper face height. Distance between soft tissue glabella and subnasale
LFH (Sn′-Me′) (mm)	Soft tissue lower face height. Distance between subnasale and soft tissue menton
*Facial proportions (hard tissue)*	
Upper face height (N-ANS) (mm)	Distance between nasion and anterior nasal spine
Lower face height (ANS-Me) (mm)	Distance between anterior nasal spine and menton
UFH (N-ANS/(N-ANS + ANS-Me)) (%)	The ratio of lower face height and total face height
LFH (ANS-Me/(N-ANS + ANS-Me)) (%)	The ratio of upper face height and total face height
Posterior face height (Co-Go) (mm)	The distance between condylion and gonion
PFH:AFH (Co-Go : N‑Me) (%)	The ratio of posterior face height and anterior face height
*Profile*	
Convexity (NA-APo) (°)	Angle between nasion, A point and pogonion
Facial angle (FH-NPo) (°)	Angle between Frankfort horizontal plane and nasion-pogonion

**Table 2:** Additional input data Ergänzende Input-Daten

Indexes	Description
Chronologic age	Grouping based on chronologic age rounded to nearest whole number
Skeletal age	Grouping based on the cervical vertebral maturation (CVM) method
Sex at birth	Grouped by male or female
Chief complaint (CC)	Grouping “Facial esthetic in the CC”, “Appearance of teeth in the CC”, and “Functional issues in the CC”, “Other”
Maxillary crowding	Grouped by amount of crowding, none, mild: 1–3 mm, moderate: 4–6 mm, severe: 7–9 mm, very severe: > 9 mm
Mandibular crowding	Grouped by amount of crowding, none, mild: 1–3 mm, moderate: 4–6 mm, severe: 7–9 mm, extremely severe: > 9 mm
Molar classification	Grouping class I, end-on class III, full step class III, beyond full step class III

### Statistical analyses

Bland–Altman plots, intraclass correlation coefficients (ICCs), and standard deviation of the repeated measurements were calculated for each cephalometric measurement. Using the test set, the success rate of the artificial neural network model was estimated, along with a 95% confidence interval (CI). To predict surgical cases, we trained a binary classifier using two different methods: random forest (RF) and logistic regression (LR).

These two machine learning algorithms were chosen as representative examples of the broader category of techniques that they belong to. RF is a non-parametric classifier and operates as an ensemble of decision trees, where each decision tree in the ensemble is considered *a weak learner *[22]. It is inspired by the fact that a large number of poorly correlated weak learners can outperform an individual constituent learner when operated as a committee. Classification in RF is performed by majority voting. The key component of the RF algorithm is the diversity of the individual models. To create a set of poorly correlated models, RF uses a random subset of features to create decision trees. The smaller the number of features selected, the less the correlation among individual models will be. However, if too few features are selected, then more trees will be needed, which will in turn increase the computational cost of training. LR belongs to the broader category of discriminative classifiers. Unlike other discriminative classifiers, LR uses a probabilistic discriminative model and can perform classification and feature selection at the same time when a 1-norm regularizer is used to optimize the discriminant vector. LR optimizes a linear hyperplane to maximize the joint posterior probabilities of training examples. As the decision surface between two classes is constrained to be linear, LR in general has very good generalization properties and is less likely to overfit the training data compared to other more complex algorithms such as artificial neural networks (ANNs) or nonlinear support vector machines (SVMs) [23] that can generate highly nonlinear decision boundaries. Confidence of a classification decision can be readily interpreted by the posterior probabilities which LR generates during testing. Hyperparameters of each classifier were tuned on the training set by 10-fold cross validation to maximize the area under the receiver operating characteristics (ROC) curve (AUC).

## Results

### Descriptive statistics

Descriptive statistics including mean, standard deviation, minimum and maximum values for the cephalometric input data are given in Table 3.

**Table 3:** Descriptive statistics of the variables Deskriptive Statistik der Variablen

	Surgery				Non-surgery			
	Mean	SD	Min	Max	Mean	SD	Min	Max
*Maxilla to cranial base*								
SNA (°)	79.06	4.39	68.80	90.30	78.06	4.28	69.30	93.90
SN-palatal plane (°)	8.39	4.03	0.40	17.00	7.78	3.54	−2.50	18.70
Occlusal plane to SN (°)	14.08	5.36	0.50	23.40	14.87	4.83	1.50	29.40
A‑N perpendicular (mm)	−1.15	4.81	−11.00	12.20	−2.15	3.88	−15.00	11.40
*Mandible to cranial base*								
SNB (°)	83.52	4.02	74.70	95.00	80.45	4.03	71.40	94.90
SNPg (°)	84.10	3.70	75.80	95.20	81.38	4.07	73.60	96.80
FMA (FH-MP) (°)	23.73	5.75	9.40	37.10	22.36	5.70	5.70	33.70
SN—MP (°)	30.48	6.34	16.20	42.70	29.38	5.76	10.90	42.90
Mandibular plane to occlusal plane (°)	17.95	4.45	10.10	32.40	15.66	4.15	7.50	25.10
B‑N perpendicular (mm)	5.66	7.13	−9.30	22.50	0.29	6.28	−19.70	22.70
Pog‑N perpendicular (mm)	7.42	7.09	−10.60	23.50	1.98	7.01	−17.30	30.80
Y‑Axis (SGn-SN) (°)	65.39	4.15	54.30	75.10	66.34	4.08	53.30	75.40
*Maxilla to mandible*								
ANB (°)	−4.45	2.36	−11.50	−0.30	−2.38	1.58	−6.40	−0.10
Palatal plane to mandibular plane angle (PP-MP) (°)	23.66	5.70	12.60	39.10	22.74	5.15	7.80	33.30
Wits appraisal (mm)	−9.77	3.18	−19.00	0.50	−5.84	2.51	−15.90	−1.10
Maxillary length (ANS-PNS) (mm)	47.04	4.37	39.60	55.50	46.16	4.25	36.80	64.70
Mandibular length (Co-Gn) (mm)	123.35	9.75	102.20	150.60	114.21	8.46	96.60	141.40
*Cranial base*								
Ba-S‑N (°)	132.47	5.68	116.70	146.90	132.19	5.70	120.00	152.70
*Upper incisors to maxilla*								
U1-SN (°)	110.81	9.04	89.80	140.60	108.78	6.89	91.70	128.90
U1-NA (°)	31.74	8.29	13.30	50.20	30.73	6.43	16.20	47.60
U1-NA (mm)	8.73	3.17	1.90	18.40	8.37	2.40	3.20	14.00
U1-palatal plane (°)	119.20	8.32	98.40	144.30	116.56	6.44	100.10	134.00
U1 protrusion (U1-APo) (mm)	4.92	3.58	−2.00	15.60	5.86	2.63	0.00	13.20
*Lower incisors to mandible*								
L1-MP (°)	84.32	8.18	66.90	112.30	87.50	7.03	68.80	104.00
L1-NB (°)	21.36	8.56	−1.40	51.30	20.07	6.31	5.90	36.10
L1-NB (mm)	4.25	3.17	−4.60	11.80	2.96	2.38	−1.70	8.80
L1 protrusion (L1-APo) (mm)	6.55	3.58	−1.80	14.70	3.76	2.64	−2.50	11.30
Holdaway ratio	−0.19	2.50	−12.70	5.30	0.50	3.58	−20.20	13.70
*Incisors to each other*								
Interincisal angle (U1-L1) (°)	131.35	13.48	80.10	163.90	131.60	10.41	108.20	159.10
Overjet (mm)	−1.38	2.93	−12.00	3.90	2.46	1.96	−3.70	5.90
Overbite (mm)	1.34	2.55	−4.40	11.50	1.77	1.64	−4.60	4.70
*Soft tissue*								
Upper lip to E‑plane (mm)	−5.81	3.78	−13.00	4.20	−4.21	2.81	−11.50	4.10
Lower lip to E‑plane (mm)	−1.43	4.49	−8.90	9.20	−1.86	3.10	−11.10	7.30
ILG (mm)	0.82	0.69	−0.70	3.90	1.19	0.78	−0.60	5.20
Nasolabial angle (Col-Sn-UL) (°)	102.77	11.09	79.10	128.50	106.24	9.83	77.80	129.70
H‑Angle (Pg′UL-Pg′Na′) (°)	8.01	5.84	−6.80	22.10	11.00	4.30	1.10	22.30
UFH (G′-Sn′) (mm)	64.22	5.53	54.30	78.70	62.69	4.42	52.50	78.20
LFH (Sn′-Me′) (mm)	69.90	7.84	53.00	91.40	66.50	6.17	48.80	79.60
*Facial proportions (hard tissue)*								
Upper face height (N-ANS) (mm)	51.60	5.00	42.40	67.80	50.02	3.35	43.20	58.00
Lower face height (ANS-Me) (mm)	64.90	6.55	50.40	80.90	60.56	5.84	46.90	77.30
UFH (N-ANS/(N-ANS + ANS-Me)) (%)	44.32	2.32	38.60	48.50	45.30	2.03	40.20	51.20
LFH (ANS-Me/(N-ANS + ANS-Me)) (%)	55.68	2.32	51.50	61.40	54.70	2.03	48.80	59.80
Posterior face height (Co-Go) (mm)	48.18	6.88	35.50	71.30	45.03	5.93	33.50	66.90
PFH:AFH (Co-Go: N‑Me) (%)	56.98	4.43	47.40	68.70	56.62	4.17	48.70	70.50
*Profile*								
Convexity (NA-APo) (°)	−10.70	6.23	−32.70	4.00	−7.18	4.36	−17.80	2.20
Facial angle (FH-NPo) (°)	93.91	3.66	84.80	101.70	91.14	3.79	80.70	105.20

*SD* standard deviation, *Min* minimum, *Max* maximum

### Reliability analyses

Bland–Altman plots, intraclass correlation coefficient (ICC), and standard deviation of the repeated measurements were calculated for each cephalometric measurement. The ICC was used to evaluate the test–retest reliabilities of the tracings. The values were scored as follows: ICC less than 0.50, poor reliability; ICC between 0.50 and 0.75, moderate reliability; ICC between 0.75 and 0.90, good reliability, and ICC greater than 0.90, excellent reliability [24]. The ICC for each repeated measurement was greater than 0.83 for all measurements except for two soft tissue measurements, interlabial gap (0.69) and nasolabial angle (0.74), demonstrating good reliability. For the initial, blinded treatment decisions, an 85% interexaminer agreement was achieved.

### Results with RF

**Fig. 2:**
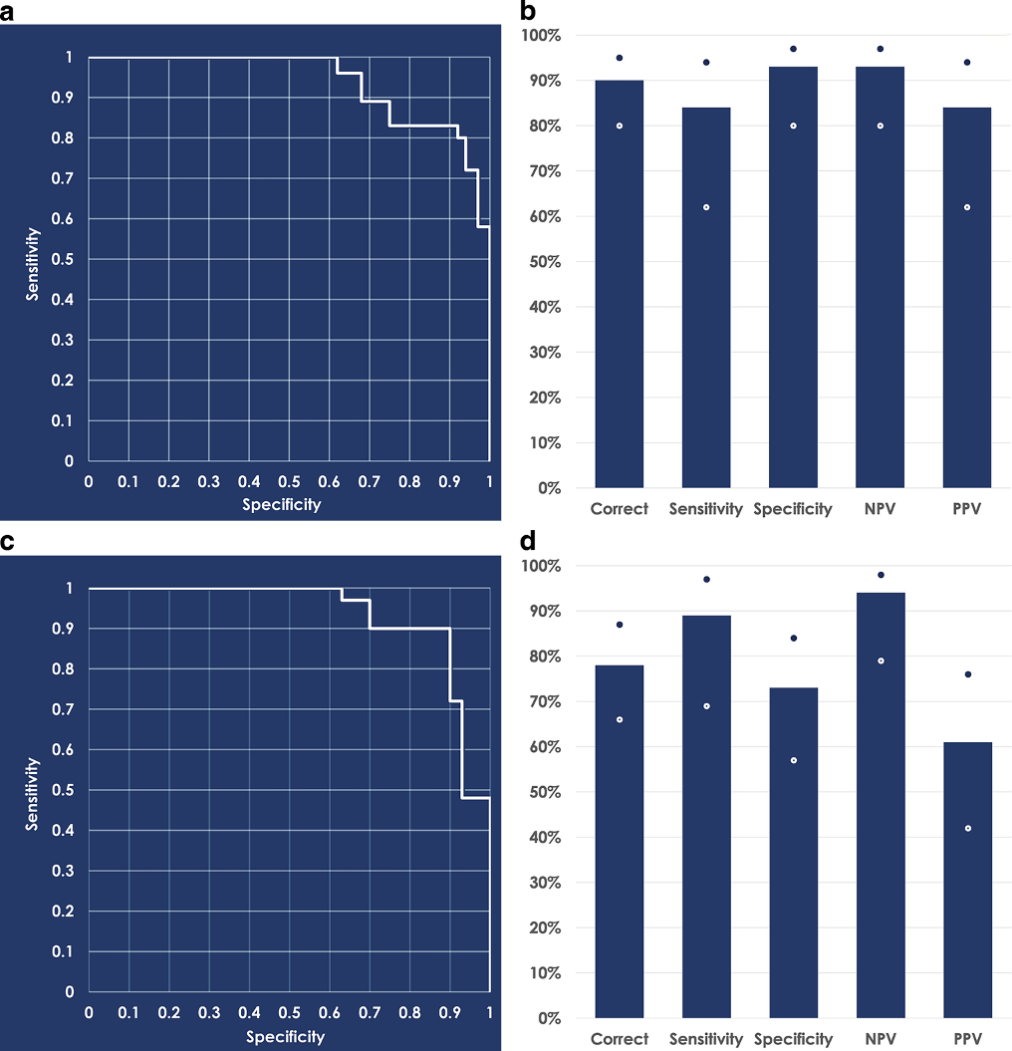
**a** Receiver operating characteristic curve obtained by the random forest classifier on the test set with an area under the curve of 0.9395; 95% confidence intervals are at [0.7908 0.9799]. **b** Classification of the random forest model with a 0.5 probability threshold. **c** Receiver operating characteristic curve obtained by the logistic regression classifier on the test set with an area under the curve of 0.937; 95% confidence intervals are at [0.8467 0.9812]. **d** Classification of the logistic regression model with a 0.5 probability threshold. *White* and *blue dots* in panels **b** and **d** represent the error bars. *NPV* negative predictive value, *PPV* positive predictive value **a** ROC(„receiver operating characteristic“)-Kurve des Random-Forest-Klassifikators für den Testsatz mit einer Fläche unter der Kurve von 0,9395; 95%-Konfidenzintervalle liegen bei [0,7908 0,9799]. **b** Klassifizierung des Random-Forest-Modells mit einer Wahrscheinlichkeitsschwelle von 0,5. **c** ROC-Kurve des logistischen Regressionsklassifikators für den Testsatz mit einer AUC von 0,937; 95%-Konfidenzintervalle liegen bei [0,8467 0,9812]. **d** Klassifizierung des logistischen Regressionsmodells mit einer Wahrscheinlichkeitsschwelle von 0,5. *NPV* negativ prädiktiver Wert, *PPV* positiv prädiktiver Wert

The number of trees in the ensemble and the number of features to subsample for training individual models are considered as tuning parameters. Another parameter that affects the performance of individual trees is the minimum number of samples required for each leaf node beyond which splitting of the node stops. These three parameters were tuned by grid optimization to maximize AUC performance for the ensemble and the final model was trained by the following values of these parameters: number of decision trees = 200, number of features to sample = 7, minimum leaf size = 5. An AUC of 0.9395 was obtained on the testing set. The 95% CIs were computed by bootstrap sampling as lower bound = 0.7908 and higher bound = 0.9799. As the lower bound was higher than 0.50, the results were statistically significantly better than a random classifier. The ROC curve is plotted in Fig. 2a. Feature importance scores were computed for the RF classifier. Although scores and rank of features varied between different runs, RF consistently found “Molar classification”, “Overjet (mm)”, and “Wits appraisal (mm)” as the top three features with the highest importance scores. RF assigned an absolute importance score of 0.05 or higher to around 80% of the 53 features available. Using a probability threshold of 0.50, the RF model was able to correctly classify cases with a 90% accuracy. The sensitivity for this model was 84% and the specificity was 93%. The RF model also showed a strong negative predictive value (NPV) of 93% and a positive predictive value (PPV) of 84% (Fig. 2b).

### Results with LR

Categorical variables (“sex at birth”, “chief complaint” and “class”) in the data were first converted into one-hot encoding vectors. With this extension the number of features increased from 53 to 60. All feature values were normalized to between 0 and 1. A regularization constant that adjusted the tradeoff between regularization and empirical error was set to 0.5. LR achieved an AUC of 0.937 on the test set. The 95% CIs were computed by bootstrap sampling as lower bound = 0.8467 and higher bound = 0.9812. As the lower bound was higher than 0.50, the results were statistically significantly better than a random classifier. The ROC curve is plotted in Fig. 2c. Only 8 of the 60 features had a non-zero weight (Table 4), which suggests that the model finds the rest of features not useful for discriminating between surgical and non-surgical cases. Using a probability threshold of 0.50, the LR model was able to correctly classify 78% of the patients. The sensitivity for this model was 89% and specificity was 73%. This model also showed a NPV of 94% and a PPV of 61% (Fig. 2d).

**Table 4:** Features selected by the logistic regression classifier with non-zero weights. Weights are optimized on normalized features Vom logistischen Regressionsklassifikator ausgewählte Merkmale mit Gewichtungen ungleich Null. Die Gewichtungen werden für normalisierte Merkmale optimiert

Features	Weights	ICC
Chief complaint: Appearance of teeth	−0.3162	–
Class 1 molar occlusion	−2.0211	–
Full step class III molar occlusion	2.5011	–
Overjet	−1.9514	0.99
ANB (°)	−0.8913	0.89
Lower incisors to mandible (°)	−0.8011	0.96
H‑angle (°)	−0.4425	0.99
Lower face height	0.1099	0.97

*ICC* intraclass correlation coefficient

## Discussion

Machine learning has been applied in many areas in dentistry for classification problems [13, 25]. The decision for surgery or non-surgery can be seen as a classification problem. Both models used in this study have previously been proven to be useful when the primary goal was outcome prediction and important interactions, or complex nonlinearities existed in a data set [26]. As RF is an ensemble of 200 decision trees and each individual tree in turn contains multiple leaf nodes (each node constitutes a rule) the results predicted by RF cannot be easily interpreted by the end user. It is often used as a black-box system, which may not present a desirable use case scenario in clinical settings. LR only used a single rule involving eight variables making it a far better interpretable model than RF. The best measurement to determine the success of each model is to assess their performance over a range of various threshold settings rather than a single operating point. Both the RF and the LR model showed high separability when classifying each patient for surgical or nonsurgical treatment with an AUC of 0.9395 and 0.937, respectively.

At a probability threshold of 0.50, RF was a little better overall at correctly classifying 90% patients for surgical or non-surgical treatment. RF was also slightly better for correctly identifying non-surgical patients with a specificity of 93%. Similarly, high levels of success were seen in other machine learning models when faced with classification between extractions [27] or surgery [21]. LR was slightly better for identifying patients requiring surgery with a sensitivity of 89%, but the tradeoff was that it was a bit worse for PPV. This shows that the model had a higher chance of identifying a patient as needing surgery when it was not recommended by the clinicians. In this study, borderline cases were defined by the 29 cases in which complete agreement was not obtained in the initial blinded treatment planning by each clinician. Of these cases, 22 were assigned to the training set and 7 were in the test set. In both models, all the cases that failed to identify the need for surgery were borderline cases. In the LR model, only 1 of the misidentified non-surgery cases was considered a borderline case. There were no borderline cases misidentified in the RF model for non-surgery. For the misidentified surgery cases, 2 of the cases in the LR model were considered borderline cases. There were 3 borderline cases misidentified in the RF model for surgery.

For this study, the input features were increased when compared to studies using similar models to expand the search for causal relationships between the independent and dependent variables [21, 27]. Many of the selected features are identical to what was found in previous studies that evaluated the surgery decision for skeletal class III patients [2, 4, 28]. More importantly, all these features play an important role in our clinical evaluation and treatment planning process. From a clinician’s perspective, the greatest indicator for orthognathic surgery is a severe anteroposterior (AP) discrepancy between both jaws. This is mostly seen with patients with a very negative ANB and Wits appraisal [28]. These patients also tend to present with a very negative overjet and severe class III molar classification.

In the most severe class III cases, patients will have an increased vertical skeletal pattern which is a combination of AP and vertical problems that typically presents with an increased lower face height [29]. These cases almost always require surgery because the movements necessary to correct the vertical relationship will worsen the AP relationship [30]. However, the advancement of skeletal anchorage systems has allowed for better non-surgical treatment success in patients with mild to moderate anterior skeletal open bites [31].

Some of the more challenging clinical decisions are on cases that could be considered borderline. The most important clinical consideration in these patients is whether the patient will be able to tolerate the dental compensation without critically effecting the esthetic result [32, 33]. The angulation of the lower incisors tends to become more compensated with camouflage treatment [34]. Patients who will more likely require surgical treatment exhibit more protrusive maxillary incisors, lingually inclined mandibular incisors, and a retrusive upper lip [30]. Generally, surgical treatment results in greater skeletal and profile changes due to the normalization of the skeletal bases [28]. The Holdaway H angle can be used to assess the balance of the lip profile to the rest of the face to determine an acceptable treatment goal for a surgical versus non-surgical approach [35]. Eslami et al. showed that the Holdaway H angle and the Wits appraisal can be used as critical diagnostic features to correctly classify 81% of patients when determining a treatment decision [4]. In another study by Stellzig-Eisenhauer et al., 92% of the patients were correctly classified with the Wits appraisal being the most decisive parameter [5]. The Holdaway H angle alone has been used to successfully classify 87% of patients [2].

## Limitations and future directions

This study was designed as a feasibility study to demonstrate the possibility of using machine learning with cephalometric and demographic data and was limited by the sample size available during the time the study was conducted. However, even with the relatively small training sample, the method was found to be successful at classifying patients in the test sample. Further follow-up studies with bigger data will help to improve the accuracy of the algorithm and allow these models to serve as another tool for orthodontists that can be used to aid in the treatment planning of surgery cases. Furthermore, adding a larger patient sample size will allow future studies to include the treatment decisions of a greater variety of experienced clinicians to incorporate differences in treatment philosophies to help refine the algorithm and shed more light on the borderline cases. Future studies should also incorporate diagnostic variables associated with the transverse dimension of occlusion which has been previously shown to improve the success rate of the model [6].

## Conclusions

This study shows that logistic regression and random forest machine learning models can be used to generate accurate and reliable algorithms to successfully classify up to 90% of patients in the treatment planning of class III orthognathic surgery. The features selected by each algorithm coincide with the clinical features that we as clinicians weigh heavily when determining a treatment plan for these patients. This study further supports that overjet, Wits appraisal, lower incisor angulation, and Holdaway H angle can be used as strong predictors in assessing a patient’s surgical needs.
